# The distinct plastid genome structure of *Maackia fauriei* (Fabaceae: Papilionoideae) and its systematic implications for genistoids and tribe Sophoreae

**DOI:** 10.1371/journal.pone.0173766

**Published:** 2017-04-11

**Authors:** In-Su Choi, Byoung-Hee Choi

**Affiliations:** Department of Biological Sciences, Inha University, Incheon, Republic of Korea; Consiglio Nazionale delle Ricerche, ITALY

## Abstract

Traditionally, the tribe Sophoreae *sensu lato* has been considered a basal but also heterogeneous taxonomic group of the papilionoid legumes. Phylogenetic studies have placed Sophoreae *sensu stricto* (*s*.*s*.) as a member of the core genistoids. The recently suggested new circumscription of this tribe involved the removal of traditional members and the inclusion of Euchresteae and Thermopsideae. Nonetheless, definitions and inter- and intra-taxonomic issues of Sophoreae remain unclear. Within the field of legume systematics, the molecular characteristics of a plastid genome (plastome) have an important role in helping to define taxonomic groups. Here, we examined the plastome of *Maackia fauriei*, belonging to Sophoreae *s*.*s*., to elucidate the molecular characteristics of Sophoreae. Its gene contents are similar to the plastomes of other typical legumes. Putative pseudogene *rps16* of *Maackia and Lupinus* species imply independent functional gene loss from the genistoids. Our overall examination of that loss among legumes suggests that it is common among all major clades of Papilionoideae. The *M*. *fauriei* plastome has a novel 24-kb inversion in its large single copy region, as well as previously recognized 50-kb and 36-kb inversions. The 36-kb inversion is shared by the core genistoids. The 24-kb inversion is present in the eight genera belonging to three tribes: Euchresteae, Sophoreae *s*.*s*., and Thermopsideae. The phylogenetic distribution of this 24-kb inversion strongly supports the monophyly of members of Sophoreae *s*.*s*. with Euchresteae and Thermopsideae. Hence, it can be used as a putative synapomorphic characteristic for the newly circumscribed Sophoreae, including Euchresteae and Thermopsideae. However, plastome conformation suggests a slightly narrower taxonomic group because of heterogeneous results from *Bolusanthus* and *Dicraeopetalum*. The phylogenetic analysis, based on plastome sequences from 43 legumes, represents well our understanding of legume systematics while resolving the genistoid clade as a sister group to an Old World clade. It also demonstrates the value that plastomes are powerful marker for systematic studies of basal papilionoid legumes.

## Introduction

Fabaceae (legumes) is the third largest angiosperm family, with approximately 751 genera and 20,000 species [1–2]. Three subfamilies—Mimosoideae, Papilionoideae, and Caesalpinioideae—are typically recognized although the first two are generally placed within the latter, which is paraphyletic. Papilionoideae is the most diverse and includes economically important legume crops. Recent comprehensive molecular phylogenies [3–4] have provided strong support for the relationships and composition of many major Papilionoideae clades. However, a systematic understanding is lacking for “basal papilionoids” [5] or “early-branching papilionoids” [3], which include tribes Swartzieae, Sophoreae, Dipterygeae, and Dalbergieae [2]. In particular, the polyphyletic tribe Sophoreae is in a systematics state of flux.

**Fig 1:**
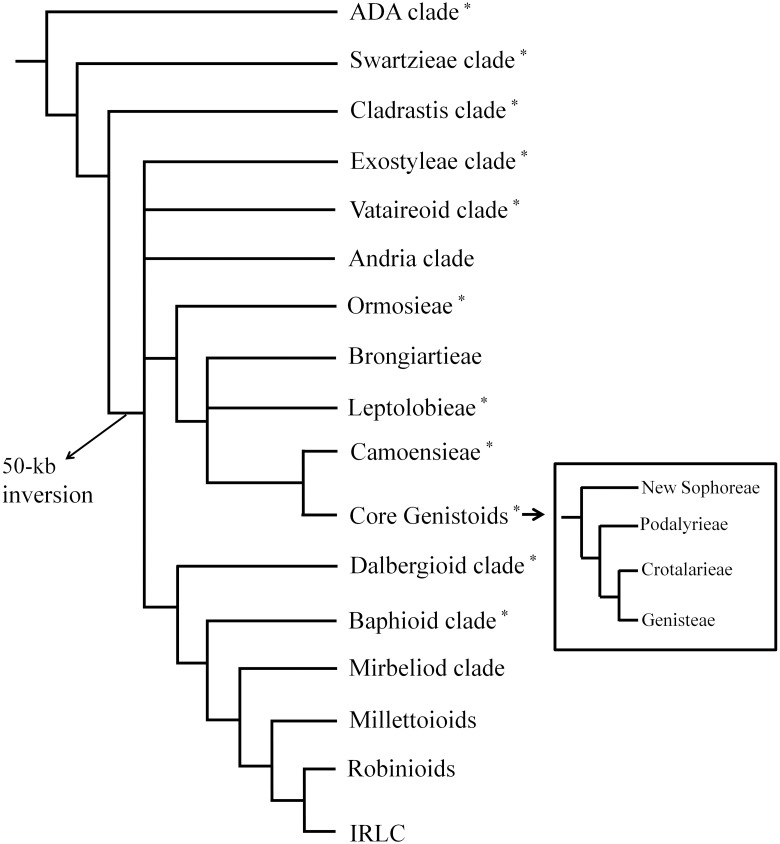
Simplified schematic phylogeny of Papilionoideae, originally published as Figure 1 in Cardoso et al. [4]. Phylogenetic positions are shown for new Sophoreae (incl. Euchresteae and Thermopsideae). *; evolutionary group that includes some former members of Sophoreae *s*.*l*. (*sensu* Polhill [6–7]).

The taxonomic circumscription of tribe Sophoreae has been debated during last several decades. The traditional tribe Sophoreae *sensu lato* (*sensu* Polhill; hereafter, ‘Sophoreae *s*.*l*.’) has been circumscribed as a vast and heterogeneous assemblage of taxa that are considered basal papilionoid legumes [6–7]. This has led to taxonomic disputes about how Sophoreae relates to early-branching papilionoid tribes [8–10]. Modern molecular phylogenies [3–5, 11–13] have shown that Sophoreae *s*.*l*. is indeed a polyphyletic group comprising genera [Sophoreae *pro parte* (*p*.*p*.)] that are scattered throughout the phylogenetic tree of the Papilionoideae (Fig 1). Meanwhile, as established by Crisp et al. [14], Sophoreae *sensu stricto* (*s*.*s*.) is considered a member of the core genistoids that include the tribes of Genisteae, Crotalarieae, Euchresteae, Podalyrieae, and Thermopsideae. Recently, Cardoso et al. [4] suggested a new circumscription of Sophoreae (hereafter, ‘new Sophoreae’) that includes 122 species of 14 genera that were formerly treated as Sophoreae *s*.*s*. (*Ammodendron* Fisch. ex DC., *Ammothamnus* Bunge, *Bolusanthus* Harms, *Dicraeopetalum* Harms, *Maackia* Rupr. & Maxim, *Platycelyphium* Harms, *Salweenia* Baker f., and *Sophora* L.), Euchresteae (*Euchresta* Benn.), and Thermopsideae (*Ammopiptanthus* S.H. Cheng, *Anagyris* L., *Baptisia* Vent., *Piptanthus* Sweet, and *Thermopsis* R. Br. ex W.T. Aiton). This new circumscription involved huge rearrangement of genera of Sophoreae *s*.*l*. and inclusion of all genera belonging to Euchresteae and Thermopsideae except *Pickeringia* Nutt. ex Torr. & A. Gray. However, their taxon sampling did not cover all genera of new Sophoreae (nine of 14 genera were included) and other studies [15–16] demonstrated that most Thermopsideae genera could also be a monophyletic group independent from Sophoreae *s*.*s*. Moreover, new Sophoreae lacks morphological criteria that can define it in the core genistoids. Thus, its taxonomic boundary remains unclear.

The plastid genome (plastome) is generally conserved among seed plants based on its quadripartite architecture [a pair of inverted repeats (IRs), small single copy (SSC) region, and large single copy (LSC) region], gene content, and order [17]. Plastomes are also generally free of recombinants, maternally inherited, and have a slow rate of evolution [18–19]. Thus, the sequence variations associated with a plastome are useful tools for studies that are phylogeographic [20–23], phylogenetic [4, 24–26], or phylogenomic [27–30] in nature. For example, within the legume phylogeny, IR losses (IRLC; Inverted Repeat-Lacking Clade); large inversions such as a 50-kb inversion (valid for all papilionoid tribes except some members of Swartzieae, Sophoreae *s*.*l*., and Dipterygeae); and gene and/or intron losses serve as important taxonomic characters [17, 31]. For genistoids, a 36-kb inversion which is embedded within 50-kb inversion and caused by flip-flop recombination from short repeat sequences of the *trnS* (GGA) and *trnS* (GCU) regions has been proposed as proof of molecular synapomorphy among a core genistoid clade [32].

Morphologically Sophoreae *s*.*l*. has been a "tribe of convenience" rather than a natural group that is clearly distinctive from both Caesalpinioideae and Papilionoideae [6]. Its non- or imperfect papilionaceous flower, with 10 free stamens, has been considered a taxonomically significant characteristic [6, 33]. However, taxa with more specialized floral features are now dominant element of new Sophoreae *sensu* Cardoso et al. [4]. Although such morphological characters can provide critical and fundamental evidence when determining taxonomic boundaries, convergent evolution of their morphology is often problematic, especially at higher taxonomic levels [34]. Therefore, it is difficult to elucidate evolutionary relationships and delimit taxonomic boundaries based only on morphology [35]. The distinct plastome characteristic has been used as a powerful marker for systematic evaluations of legumes [17, 31]. Thus, plastome studies could be promising for testing the validity of the new Sophoreae.

Here, we sequenced and analyzed the first plastome of Sophoreae *s*.*s*. from *Maackia fauriei* (H. Lév.) Takeda, a large tree endemic to Jeju Island, Korea. Although a single plastid genome sequence is not sufficient to answer all of the taxonomic problems surrounding Sophoreae *s*.*l*., we did identify a distinct plastome organization from *M*. *fauriei* and then screened those features in representatives of genistoid and Sophoreae *s*.*l*. We also reconstructed a legume phylogeny based on the plastome sequences found in our current study as well as those reported recently by other researchers.

## Materials and methods

### Ethics statement

*Maackia fauriei* is not an endangered or protected species in Korea. We did not collect plants from the protected areas that required permission.

### Plastid genome sequencing

Leaves of *Maackia fauriei* were collected from Jeju Island, Korea, and preserved with silica gel. Voucher specimen (J.Y. Lee & I.S. Choi 1208046) were deposited in the Herbarium of Inha University (IUI), Incheon, Korea. Genomic DNA was extracted from the dried tissues by a protocol that utilized the DNeasy Plant Mini Kit (Qiagen, Seoul, Korea). The quantity and quality of the extracted genomic DNA were assessed by gel electrophoresis and spectroscopy with a NanoDrop ND-1000 (NanoDrop Technologies, Wilmington, DE, USA). The gDNAs were then fragmented into 500-bp segments for library construction per the manufacturer’s instructions (Illumina Inc., San Diego, CA, USA), and were sequenced using the Illumina HiSeq 2000 System (Illumina Inc.) at the BML Co. in Daejeon, Korea.

### Plastid genome assembly

The sequencing run produced 25,318,060 paired-end reads (101 bp each). Poor-quality sequences were removed by Trimmomatic 0.32 [36]. To isolate plastid-related reads and assemble the plastome, we largely followed the method described by Wang and Messing [37]. Briefly, the paired-end reads were mapped on the reference genome of *Lupinus luteus* L. (GenBank Accession Number NC_023090) using Geneious ver. 7.1.3 (Biomatters Ltd., Auckland, NZ). The mapped reads were then reassembled *de novo* using Geneious and the generated plastome contigs were re-aligned to the plastome of *L*. *luteus* to identify gaps among the contigs. This generated four contigs (two for LSC and two each for the SSC and IR regions) with a total length of approximately 154 kb. To fill those gaps, we used purified DNA and performed polymerase chain reactions (PCRs) with primers designed *via* Primer3 [38].

### Gene annotation

The complete plastome for *M*. *fauriei* was annotated using DOGMA [39] and Geneious. The tRNAs were confirmed by tRNAscan-SE [40]. Other protein-coding regions were checked based on data from the NCBI (http://blast.ncbi.nlm.nih.gov/), and manual corrections were made for the start and stop codons. Particular gene features of the plastome were illustrated with the Web-based tool OGDraw [41].

### Investigation of *rps16* among legume plastomes

The loss of *rps16* was investigated in complete plastomes from 33 legume species (Table 1). Each *rps16* gene was extracted from the available sequenced genomes and sequences were aligned by MUSCLE 3.8.31 [42] using default parameters. We re-analyzed and categorized this gene in four ways, as in Kim et al. [43]: 1) intact gene (full-length and in-frame), 2) putative pseudogene (mutation in start or stop codon or frame shift-inducing indels), 3) truncated gene (significant deletion), and 4) complete deletion.

**Table 1:** Comparison of features from legume plastomes selected for this study.

Subfamily	Tribe	Species	Accession number	*rps16*	Total genome size (bp)
Caesalpinioideae	Cercideae	*Cercis canadensis*	KF856619	intact gene	158,995
Caesalpinioideae	Detarieae	*Tamarindus indica*	KJ468103	intact gene	159,551
Caesalpinioideae	Caesalpinieae	*Ceratonia siliqua*	KJ468096	intact gene	156,367
Caesalpinioideae	Cassieae	*Chamaecrista fasciculata*	KP126855	partial genome	
Caesalpinioideae	Caesalpinieae	*Caesalpinia coriaria*	KJ468095	intact gene	158,045
Caesalpinioideae	Caesalpinieae	*Haematoxylum brasiletto*	KJ468097	intact gene	157,728
Mimosoideae	Mimoseae	*Prosopis glandulosa*	KJ468101	intact gene	163,042
Mimosoideae	Mimoseae	*Leucaena trichandra*	NC_028733	intact gene	164,692
Mimosoideae	Mimoseae	*Desmanthus illinoensis*	KP126868	partial genome	
Mimosoideae	Ingeae	*Inga leiocalycina*	NC_028732	intact gene	175,489
Mimosoideae	Acacieae	*Acacia ligulata*	NC_026134	intact gene	174,233
Papilionoideae	Amorpheae	*Amorpha canescens*	KP126852	partial genome	
Papilionoideae	Dalbergieae	*Arachis hypogaea*	KJ468094	complete deletion	156,395
Papilionoideae	Genisteae	*Lupinus albus*	KJ468099	putative pseudogene	154,140
Papilionoideae	Genisteae	*Lupinus luteus*	NC_023090	putative pseudogene	151,894
Papilionoideae	Sophoreae	*Maackia fauriei*	KX388160	putative pseudogene	154,541
Papilionoideae	Thermopsideae	*Baptisia alba*	KP126860	partial genome	
Papilionoideae	Thermopsideae	*Baptisia bracteata*	KP126854	partial genome	
Papilionoideae	Indigofereae	*Indigofera tinctoria*	KJ468098	intact gene	158,367
Papilionoideae	Millettieae	*Millettia pinnata*	NC_016708	intact gene	152,968
Papilionoideae	Phaseoleae	*Apios americana*	KF856618	complete deletion	152,828
Papilionoideae	Phaseoleae	*Pachyrhizus erosus*	KJ468100	intact gene	151,947
Papilionoideae	Phaseoleae	*Glycine max*	NC_007942	intact gene	152,218
Papilionoideae	Psoraleeae	*Pediomelum argophyllum*	KP126866	partial genome	
Papilionoideae	Psoraleeae	*Psoralidium tenuiflorum*	KP126859	partial genome	
Papilionoideae	Phaseoleae	*Phaseolus vulgaris*	NC_009259	truncated gene	150,285
Papilionoideae	Phaseoleae	*Strophostyles leiosperma*	KP126853	partial genome	
Papilionoideae	Phaseoleae	*Vigna unguiculata*	KJ468104	intact gene	151,866
Papilionoideae	Robinieae	*Robinia pseudoacacia*	KJ468102	truncated gene	154,835
Papilionoideae	Loteae	*Lotus japonicus*	NC_002694	intact gene	150,519
Papilionoideae	Millettieae	*Wisteria floribunda*	NC_027677	putative pseudogene	130,960
Papilionoideae	Galegeae	*Glycyrrhiza glabra*	NC_024038	truncated gene	127,943
Papilionoideae	Galegeae	*Astragalus mongholicus* var. *nakaianus*	NC_028171	truncated gene	123,633
Papilionoideae	Galegeae	*Oxytropis lambertii*	KP126858	partial genome	
Papilionoideae	Cicereae	*Cicer arietinum*	NC_011163	truncated gene	125,319
Papilionoideae	Trifolieae	*Melilotus albus*	KP126850	partial genome	
Papilionoideae	Trifolieae	*Medicago truncatula*	NC_003119	complete deletion	124,033
Papilionoideae	Trifolieae	*Trifolium aureum*	NC_024035	truncated gene	126,970
Papilionoideae	Trifolieae	*Trifolium subterraneum*	NC_011828	truncated gene	144,763
Papilionoideae	Fabeae	*Pisum sativum*	NC_014057	complete deletion	122,169
Papilionoideae	Fabeae	*Lathyrus sativus*	NC_014063	complete deletion	121,020
Papilionoideae	Fabeae	*Vicia faba*	KF042344	truncated gene	123,722
Papilionoideae	Fabeae	*Lens culinaris*	NC_027152	complete deletion	123,096

### Whole-genome alignments

Other plastome sequences for relevant legumes (Table 1) were obtained from GenBank. Single IR copies were manually removed. Afterward, the complete plastome of *M*. *fauriei* and other sequences [*Tamarindus indica* L. (KJ468103), *Arachis hypogaea* L. (KJ468094), and *Lupinus luteus* (NC_023090)] were aligned using genome alignment software Mauve 2.3.1 [44] to check for inversion events.

### Survey of inversion events among genistoids and Sophoreae *s*.*l*.

To verify the distribution of 36-kb and 24-kb inversions on the phylogenetic tree, we used methods based on PCR amplifications. In all, 16 representative species were selected that belong to five tribes and 15 genera (Table 2). They roughly cover the genistoids and Sophoreae *s*.*l*. group. Those plant materials had been collected from East Asia (China, Korea, and Japan) and deposited at the IUI herbarium. Other DNA samples were obtained from the DNA Banks of the Royal Botanic Gardens, Kew (http://apps.kew.org/dnabank/homepage.html) and the University of Johannesburg (UJ), South Africa (http://acdb.co.za/index.php/dna-bank/introduction-2.html). The remaining materials needed for this study were also collected through KNRRC (Medicinal Plants Resources Bank NRF-2010-0005790), supported by the Korea Research Foundation and the Ministry of Education, Science and Technology in 2014. Vouchers were deposited at the herbarium of Gachon University (GCU). The gDNAs were extracted from silica gel-dried leaves, as described above. All PCRs were conducted with a GeneAmp^®^ PCR System 2700 Thermal Cycler (Applied Biosystems, Foster City, CA, USA) according to a program of initial denaturation that was followed by 30 cycles of 10 s at 98°C, 7 s at 58°C, and 2 min at 72°C; and then a final extension for 5 min at 72°C. Each 50-μL reaction mixture included 1 μL of genomic DNA (~ 20 ng), 1 μL each of forward and reverse primers (10 pMol), and 25 μL of PrimeSTAR HS Premix (TaKaRa, Seoul, Korea). The existence of the 36-kb inversion was tested using primer pairs designed by Martin et al. [32]. The pair of rps4-bef-F and ycf3-bef-R was used for determining its absence while ycf3-inv-F and psbI-int-R were used to detect its presence. Three primers were designed to test for the 24-kb inversion: the pair of FGA-ndhJ-F and FGA-trnF-R for its absence and the pair of FGA-ndhJ-F and FGA-trnC-R for its presence (Table 3). The PCR products were visualized on 2% agarose gels, purified by PCR quick-spin TM (iNtRON Biotechnology, Seongnam, Korea), and sequenced with an ABI 3100 Genetic Analyzer and an ABI BigDyeTM Terminator Cycle Sequencing Ready Reaction Kit (Applied Biosystems) at the Macrogen, Seoul, Korea.

**Table 2:** Taxa sampled to screen for inversions.

Taxon	Classification			Inversion events		Voucher information
	Polhill (1981 and 1994) [6–7]	Lewis et al. (2005) [1]	Cardoso et al. (2013) [4]	36-kb	24-kb	
*Cladrastis wilsonii*	Sophoreae	Sophoreae *p*.*p*.	*Cladrastis* clade	-	-	I.S. Choi & D.P. Jin 1209001 (IUI)
*Styphnolobium japonicum*	Sophoreae	Sophoreae *p*.*p*.	*Cladrastis* clade	-	-	B.H. Choi 1317 (IUI)
*Camoensia brevicalyx*	Sophoreae	Sophoreae *s*.*s*.	Camoensieae	-	-	Champluvier 5205 (K)(Kew 33888)
*Crotalaria capensis*	Crotalarieae	Crotalarieae	Crotalarieae	+	-	O. Maurin et al. OM2692 (JRAU)(UJ 05004)
*Lupinus luteus*	Genisteae	Genisteae	Genisteae	+	-	ABH 31123 (ABH)(Kew 15870)
*Bolusanthus speciosus*	Sophoreae	Sophoreae *s*.*s*.	Sophoreae	+	-	O. Maurin et al. OM240 (JRAU)(UJ 00040)
*Dicraeopetalum mahafaliense*	Sophoreae	Sophoreae *s*.*s*.	Sophoreae	+	-	D. Puy et al. 1029 (Kew 13059)
*Anagyris foetida*	Thermopsideae	Thermopsideae	Sophoreae	+	+	Crespo & E. galante 38544 (ABH)(Kew 15865)
*Baptisia australis*	Thermopsideae	Thermopsideae	Sophoreae	+	+	I.S. Choi 1405001 (IUI)
*Piptanthus nepalensis*	Thermopsideae	Thermopsideae	Sophoreae	+	+	H. Sun s.n. (GCU)(MPRBP 01013)
*Thermopsis fabacea*	Thermopsideae	Thermopsideae	Sophoreae	+	+	B.H. Choi & I.S. Choi 1406001 (IUI)
*Maackia fauriei*	Sophoreae	Sophoreae *s*.*s*.	Sophoreae	+	+	J.Y. Lee & I.S. Choi 1208046 (IUI)
*Salweenia bouffordiana*	Sophoreae	Sophoreae *s*.*s*.	Sophoreae	+	+	H. Sun s.n. (GCU)(MPRBP 01012)
*Sophora koreensis*	Sophoreae	Sophoreae *s*.*s*.	Sophoreae	+	+	J.Y. Lee & B.H. Choi s.n. (IUI)
*Sophora flavescens*	Sophoreae	Sophoreae *s*.*s*.	Sophoreae	+	+	S.G. Jung s.n. (IUI)
*Euchresta japonica*	Euchresteae	Euchresteae	Sophoreae	+	+	B.H. Choi 99078 (IUI)

For inversion events: + indicates presence of inversion; –, absence. DNA bank numbers are in parentheses after voucher information.

**Table 3:** PCR primers used for screening of inversion events.

Primer	Sequence	Source
rps4-bef-F	5′-CAATCAAATAATAGATAGTAAATGGGTTG-3′	Martin et al. [32]
ycf3-bef-R	5′-GGAATTATTCGTAATAATATATTGGCTAC-3′	Martin et al. [32]
ycf3-inv-F	5′-CGTAATAAGATATTGGCTAC-3′	Martin et al. [32]
psbI-int-R	5′-CTCTTTTCATCTTCGGATTC-3′	Martin et al. [32]
FGA-ndhJ-F	5′-CGTTCCCAATGTGCCTAT-3′	This study
FGA-trnF-R	5′-TGGTAGAGCAGAGGACTG-3′	This study
FGA-trnC-R	5′-CAAATCCTTTTTCCCCAGTT-3′	This study

### Phylogenetic analysis

A phylogenetic tree was constructed using 43 complete or partial plastomes for legumes, as collected from the GenBank database (Table 1). The outgroup included *Morus mongolica* (Bureau) C.K. Schneid. (KM491711), *Fragaria vesca* L. (NC_015206), *Castanea mollissima* Blume (NC_014674), and *Cucumis sativus* L. (DQ119058), as described by Schwarz et al. [45]. The final data set from these 47 plastomes comprised 71 conserved plastid protein-coding genes: *atpA*, *B*, *E*, *F*, *H*, and *I*; *ccsA*; *cemA*; *clpP*; *matK*; *ndhA*, *B*, *C*, *D*, *E*, *F*, *G*, *H*, *I*, *J*, and *K*; *petA*, *B*, *D*, *G*, *L*, and *N*; *psaA*, *B*, *C*, *I*, and *J*; *psbA*, *B*, *C*, *D*, *E*, *F*, *H*, *I*, *J*, *K*, *L*, *M*, *N*, *T*, and *Z*; *rbcL*; *rpl2*, *14*, *16*, *20*, *23*, *32*, *33*, and *36*; *rpoA*, *B*, *C1*, and *C2*; *rps2*, *3*, *4*, *7*, *8*, *11*, *12*, *14*, *15*, and *19*; and *ycf3*. The alignments were made with MAFFT (v. 7.017) and default parameters. Poorly aligned regions were either refined or deleted using Geneious. A maximum likelihood (ML) analysis of the complete, final alignment of all taxa was conducted with RAxML Blackbox [46], using the gamma model of rate heterogeneity and an ML search.

## Results

### Plastome sequence of *Maackia fauriei* and variation in *rps16* loss among legumes

**Fig 2:**
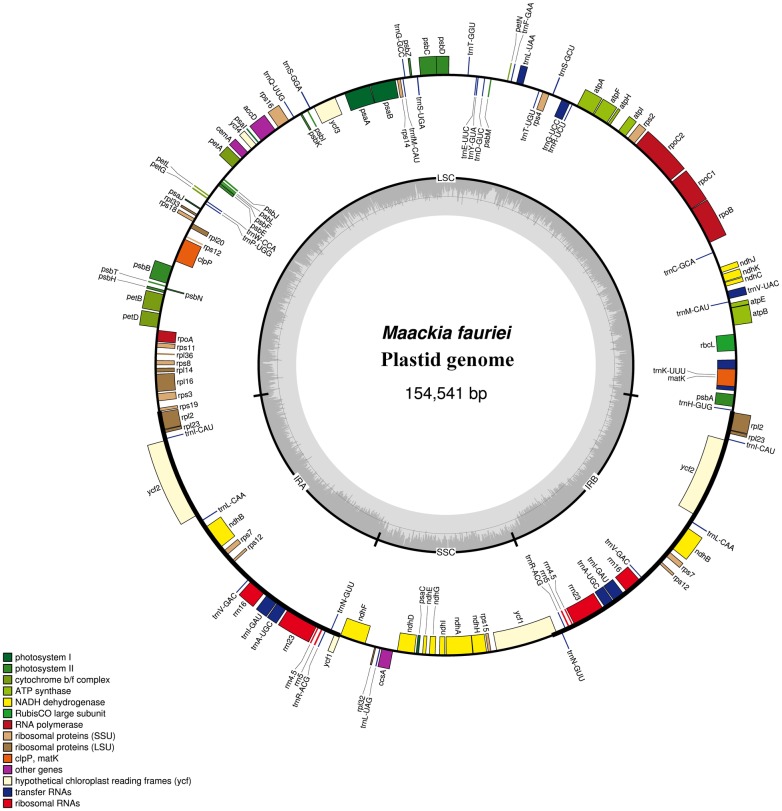
Map of *Maackia fauriei* plastome. Genes on outside of outer circle are transcribed in clockwise direction; those on inside of outer circle are transcribed in counterclockwise direction. Functional categories of genes are color-coded. Dashed area in inner circle indicates GC content of plastid genome.

The complete plastome of *Maackia fauriei* (GenBank Accession No. KX388160) is 154,541 bp long and has two IR regions (25,494 bp each) that are separated by LSC and SSC regions of 85,140 bp and 18,413 bp, respectively (Fig 2). Approximately 57.9% of the sequence is composed of protein-coding regions while the remaining 42.1% contains non-coding sequences that include introns and intergenic spacers (IGS). The AT and GC contents are 63.5% and 36.5%, respectively. Among the 135 recognized genic features are four unique rRNAs, 31 tRNAs, and 76 protein-coding genes. The *rpl22* loss, which is typical legume plastomes, is shared by *Maackia*. The *rps16* is a putative pseudogene caused by “AAAC” duplication in exon2 that results in a frame shift mutation and internal stop codon.

**Fig 3:**
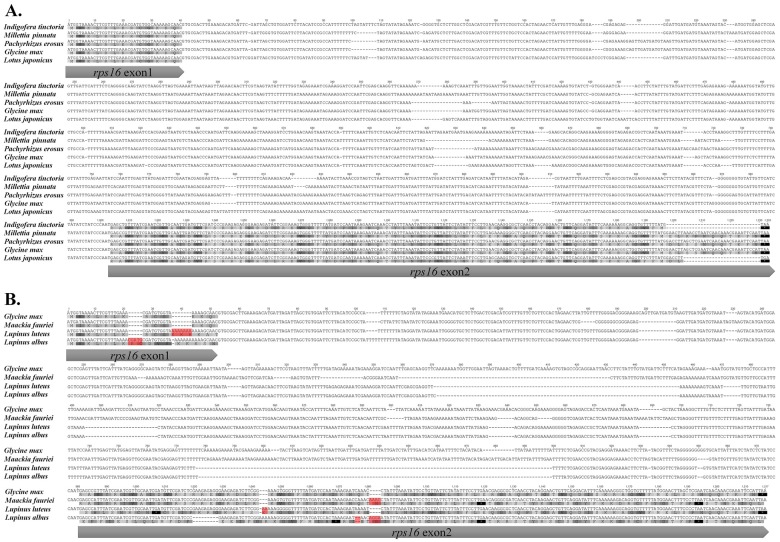
Sequence alignment of intact genes and putative pseudogenes of *rps16*. A, intact *rps16* in papilionoids; B, putative pseudogene *rps16* in genistoids, with comparison to *Glycine max* (L.) Merr. Frameshift-inducing indels are shaded in red.

We also investigated the loss of *rps16* among 33 complete legume plastomes (Table 1) and found that the gene was intact in 15 species, a putative pseudogene in four species, truncated pseudogene in eight species, and deleted in six species. All of the Caesalpinioideae and Mimosoideae samples contained intact *rps16*. The types of losses varied for *rps16* and were restricted to Papilionoideae. We considered the *rps16*s from *Lupinus* L. species as putative pseudogenes similar to that of *M*. *fauriei* due to several indel events on the exons and an approximately 200-bp deletion of intron sequences (Fig 3).

### The 36-kb and 24-kb inversion events and their phylogenetic distribution among genistoids and Sophoreae *s*.*l*.

**Fig 4:**
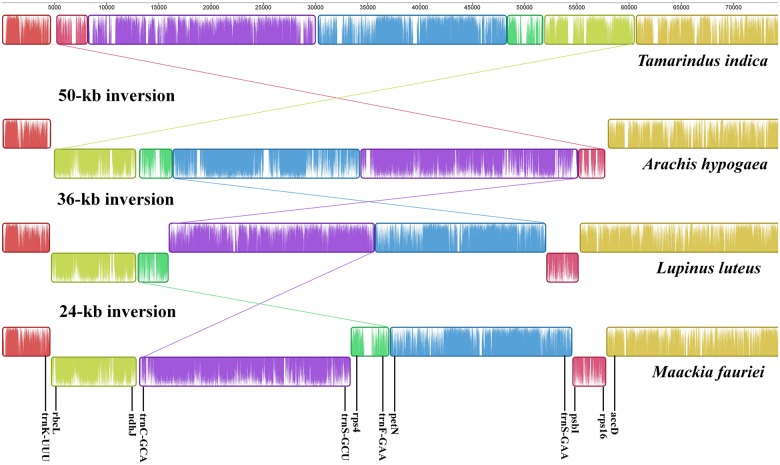
Plastome alignment of LSC regions from *Maackia fauriei* and other legume species.

The legume species used for our comparisons revealed only subtle differences in their gene content. However, the gene order for *Maackia fauriei* did not resemble that of any other species. To trace these evolutionary changes, we selected four legume plastomes based on the results of phylogenetic analysis. Our Mauve alignment of the LSC region among *M*. *fauriei* and other legumes generated seven locally collinear blocks (LCBs) that represented a homologous region without rearrangement (Fig 4). These LCBs indicated that *M*. *fauriei* experienced at least three inversion events: 1) a 50-kb inversion between IGSs near *accD* and *trnK* (UUU), which is the major landmark in most papilionoid legume plastomes; 2) a 36-kb inversion situated between the 29-bp identical sequences of *trnS* (GGA) and *trnS* (GCU), a feature shared by *Lupinus* among core genistoids and *Robinia* L. in the robinioids; and 3) a 24-kb inversion embedded in the 50-kb inversion region, an event that is newly discovered from the *M*. *fauriei* plastome and which occurs between IGSs near *trnC* (GCA) and *trnF* (GAA).

**Fig 5:**
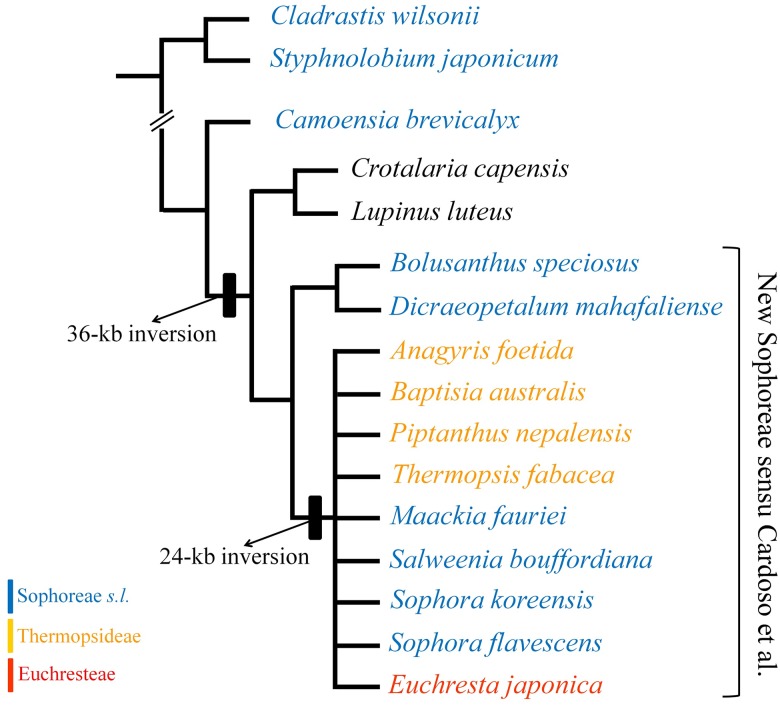
Phylogenetic distribution of 36-kb and 24-kb inversion events in plastomes, as surveyed in our study. All taxa and their classifications are detailed in Table 2. Arrangement of taxa is based on current understanding about phylogenies of these taxa [3–4, 14–16, 25, 47].

To verify the distribution of these 36-kb and 24-kb inversions on our phylogenetic tree, we used PCR-screening with 16 representative species belonging to five tribes—14 genera of genistoids and the Sophoreae *s*.*l*. group. All sequences were deposited in GenBank (Accessions KX430180 through KX430211). This strategy demonstrated that three genera—*Cladrastis* Raf., *Styphnolobium* Schott, and *Camoensia* Welw. ex Benth.–did not have those inversions (Table 2 and Fig 5). However, the 36-kb inversion was distributed within the five tribes of core genistoids while the 24-kb inversion was shared by eight genera in three genistoid tribes—Euchresteae, Sophoreae *s*.*s*., and Thermopsideae. Two other genera from Sophoreae *s*.*s*.—*Bolusanthus* and *Dicraeopetalum*—did not share the 24-kb inversion and had only 36-kb inversions in their plastomes.

### Phylogenetic analysis

**Fig 6:**
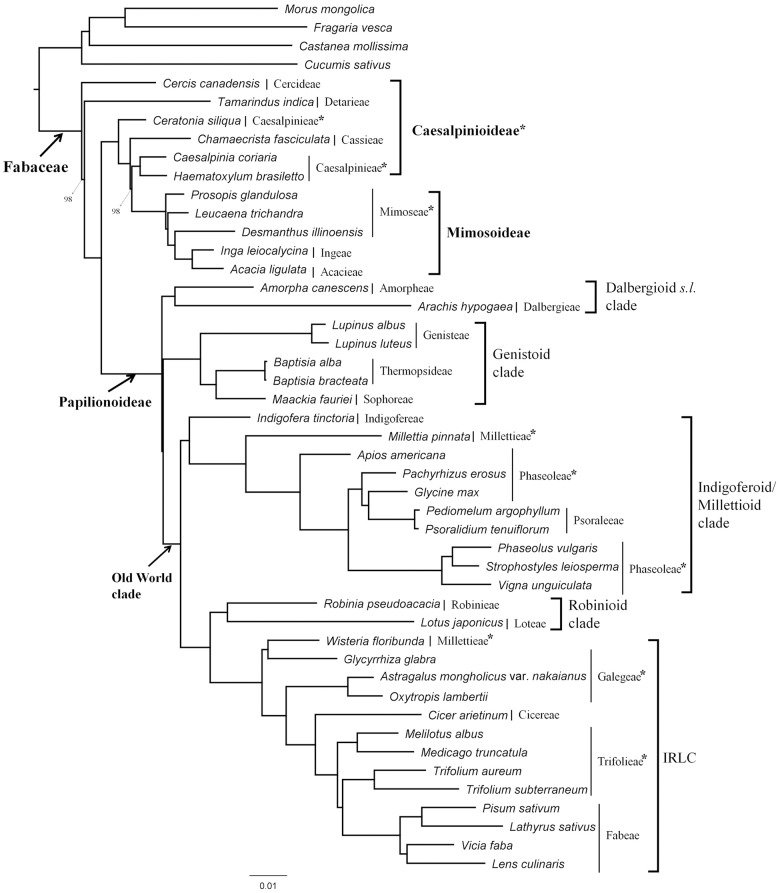
Maximum likelihood phylogeny tree of legume species inferred by RAxML Blackbox. Phylogenetic relationships among taxa were generated from concatenated matrix of 71 protein-coding genes with combined aligned length of 53,307 characters. Values at nodes are shown when bootstrap support values are not 100%. Asterisks mark taxa that are not monophyletic.

Our data set for the phylogenetic analysis contained 71 protein-coding genes from 47 taxa, including 43 legumes and four outgroups. This accounted for 53,307 nucleotide positions. The ML analysis of those 47 taxa resulted in a single best-scoring tree with—lnL = 358979.245. Bootstrap analyses indicated that, except for two nodes outside of Papilionoideae, all nodes were supported by values of 100%. The ML phylogeny indicated that Caesalpinioideae was basal and paraphyletic and that Mimosoideae and Papilionoideae formed monophyletic groups nested within the Caesalpinioideae (Fig 6). We also recognized five sub-clades of papilionoids, i.e., dalbergioid *s*.*l*., genistoid, indigoferoid/millettioid, robinioid, and IRLC. The dalbergioid *s*.*l*. was the first diverging clade, followed by genistoids. Branch lengths were very short between nodes of dalbergioid *s*.*l*. and the genistoids. *Maackia fauriei* was nested within the genistoids with Thermopsideae (*Baptisia*) and Genisteae (*Lupinus*). The remaining three sub-clades formed the Old World clade, with indigoferoid/millettioid being the first to branch, followed by robinioid and IRLC. Among the 22 tribes of legumes examined here, six (Caesalpinieae, Mimoseae, Millettieae, Phaseoleae, Galegeae, and Trifolieae) were deemed non-monophyletic. Overall, the relationships within legumes were in agreement with recently described phylogenies [2–4]. The exception was the genistoids, which were resolved as a sister group to the Old World clade.

## Discussion

### Characteristics of the *Maackia fauriei* plastome and independent *rps16* loss in genistoids

The complete plastome of *Maackia fauriei* includes four rRNA, 31 tRNA, and 76 protein-coding gene species. As with the other legume plastomes [45], this genome lacks *rpl22*, which is transferred to the nucleus [48]. Moreover, this genome has sequences consistent with the presence of a putative pseudogenized *rps16*. Using slot blot hybridization with various legume species, Doyle et al. [49] found multiple and independent *rps16* losses among papilionoids. Schwarz et al. [45] suggested that these losses have independently occurred at least five times in Papilionoideae, but not in genistoids. However, our analysis of *rps16* loss patterns demonstrated that the plastomes of genistoids also experienced independent loss events. We categorized *rps16* as an intact gene, putative pseudogene, truncated gene, or complete deletion (Table 2). The plastomes of Caesalpinioideae, Mimosoideae, and some of the Papilionoideae (i.e., *Indigofera* L., *Millettia* Wight & Arn., *Pachyrhizus* Rich. ex DC., *Glycine* L., and *Lotus* L.) have intact *rps16*s that have no internal indels in the exons except at the ends. Truncations occur sporadically in papilionoid legumes and are largely attributed to the severe deletion of exon2. Exon1 of *rps16* is relatively conserved in papilionoid legumes, except for complete deletions, as observed with *Arachis* L., *Apios* Fabr., and most members of Fabeae. Putative pseudogenes are relatively rare and only observed within *Wisteria* Nutt. and genistoid species (Fig 3). The *rps16* of *Lupinus* species has previously been annotated as in-frame and is regarded as an intact gene [32, 45]. However, it does not seem to be functional because of severe frame-shifts and deletions in the intron revealed by our investigation. Overall, *rps16* losses exist for the five major clades—dalbergioid *s*.*l*., genistoid, indigoferoid/millettioid, robinioid, and IRLC—and all feature 50-kb inversions in their plastomes.

### Systematic implications of plastome inversions in genistoids and Sophoreae

The genistoid is an informal taxonomic group of legumes that are characterized by quinolizidine alkaloids and a base chromosome number of n = 9 [5, 12, 50]. The tribes Genisteae, Crotalarieae, Euchresteae, Podalyrieae, Sophoreae *s*.*s*., and Thermopsideae are consistently resolved as a monophyletic group within genistoids, and are considered the core genistoids [3–4, 12, 14]. We found evidence here that the plastome of *Maackia fauriei*, belonging to Sophoreae *s*.*s*., has undergone three inversion events (50-kb, 36-kb, and 24-kb) based on our Mauve alignment with other legume species (Fig 4). Martin et al. [32] examined the distribution of the 36-kb inversion and their taxon sampling included two species outside of the genistoids [i.e., *Cladrastis lutea* (Michx.) K. Koch and “*Sophora japonica*”], plus nine genera of core genistoids belonging to one genus within Thermopsideae (*Thermopsis*) and eight genera within Genisteae (*Argyrolobium* Eckl. & Zeyh., *Lupinus*, *Chamaecytisus* Link, *Laburnum* Fabr., *Retama* Raf., *Ulex* L., *Echinospartum* Fourr., and *Genista* L.). They have argued that the 36-kb inversion is potentially a molecular synapomorphy for core genistoids. In the current study, we examined 16 representative species (Table 2) that included five core genistoid tribes (Crotalarieae, Euchresteae, Genisteae, Sophoreae *s*.*s*., and Thermopsideae), and also Sophoreae *s*.*l*., that are outside of core genistoid [*Cladrastis wilsonii* Takeda, *Styphnolobium japonicum* (L.) Schott (= *Sophora japonica* L.), and *Camoensia brevicalyx* Benth.]. The 36-kb inversion is absent from species outside of the core genistoid, including those in its putative sister group, *Camoensia*. The genus *Camoensia* was once treated as a core genistoid [3] because it was thought to be a member of Sophoreae *s*.*s*. [35]. However, Cardoso et al. [4] resurrected *Camoensia* as being in the monotypic tribe Camoensieae and treated it as part of a sister group instead. Our findings that differ from those of Martin et al. [32] are the plastome conformation and classification for *Styphnolobium japonicum* (= *Sophora japonica*) which is clearly not in the core genistoid group [5, 50–53]. Instead, our research indicates that *S*. *japonicum* lacks the 36-kb inversion, similar to its close relative *Cladrastis*. Three genera—*Cladrastis*, *Pickeringia*, and *Styphnolobium*—are currently known as being free from plastome rearrangements and forming a sister clade to vast papilionoid legume taxa marked by 50-kb inversion [2, 4]. Thus, this contrast with the report by Martin et al. [32] might be an outcome of mistakes made during the earlier experimental process rather than being an independent parallel-inversion event at the intra-species level. Unlike the case of *Styphnolobium*, the 36-kb inversion has occurred independently for the genus *Robinia*, which is thought to be distantly related evolutionarily but includes 50-kb inversion in its plastome [45]. Thus, the possible existence of a parallel 36-kb inversion from evolutionarily close taxa means that its value in molecular synapomorphy is questionable among genistoids. Additional plastome sequences from other early-diverging papilionoid legumes are warranted. Nevertheless, the absence of such an inversion in *Camoensia* but its presence in all core genistoid tribes tested here is distinct evidence that supports the recent research on core genistoids [4].

The Sophoreae *s*.*l*. have largely included the taxa with least specialized flowers that are similar to those within Caesalpinioideae [6–7] even though its type genus, *Sophora*, has more specialized and papilionaceous floral characteristics than other basal papilionoid legumes [33]. Recent molecular phylogenetic studies [3–5, 11–13] have also demonstrated that Sophoreae *s*.*s*. is more closely related to tribes of Euchresteae and Thermopsideae, which share specialized papilionaceous flowers. Likewise, our study demonstrated that the Sophoreae *s*.*s*. has a more specialized plastome organization (Fig 4) that has resulted from the combination of three inversion events (i.e. 50-kb, 36-kb, and 24-kb). This characteristic is also shared with Euchresteae and Thermopsideae. The new Sophoreae *sensu* Cardoso et al. [4] includes the merger of Euchresteae and Thermopsideae into Sophoreae and removal of a large number of genera that were traditionally considered members of Sophoreae. The formal taxonomic revision for new Sophoreae remains to be done and a synapomorphic characteristic for this group was lacking. Hence, the distinct plastome organization revealed from our study could be a putative molecular synapomorphy for new Sophoreae. In contrast, it is also conceivable that a 24-kb inversion occurred at distantly related lineages, as was the case for 36-kb parallel inversions [45]. However, the plastome conformation of Sophoreae *s*.*s*. is not likely to be a homoplasious characteristic. Researchers have assumed that the 36-kb inversion (39-kb inversion for *Robinia*) was mediated by flip-flop recombination between the conserved 29-bp repeats of two *trnS* genes [32, 45]. By comparison, the breakpoints of 24-kb inversion are IGSs near *trnC* (GCA) and *trnF* (GAA), which are not conserved sequences. Furthermore, the plastome conformation of Sophoreae *s*.*s*. was not formed by a single inversion but, combinationally and sequentially, through three independent inversion events. Hence, a 24-kb inversion seems to be unique to a monophyletic group that comprises Sophoreae *s*.*s*. and its closely related tribes Euchresteae and Thermopsideae.

Two genera, *Bolusanthus* and *Dicraeopetalum*, that are distributed in the Afro-Madagascan region, have long been included in Sophoreae (whether *sensu stricto* or *lato*) [4, 6–7, 35]. However, we found a heterogeneous plastome conformation (i.e., lack of a 24-kb inversion) when those two were compared with other Northern Hemisphere genera (*Anagyris*, *Baptisia*, *Piptanthus*, *Thermopsis*, *Maackia*, *Salweenia*, *Sophora*, and *Euchresta*) (Table 2 and Fig 5). The presence of a 36-kb inversion in *Bolusanthus* and *Dicraeopetalum* and recent phylogenetic evidence [3–4] indicate that they are members of the core genistoid clade. However, inclusion of either into the new Sophoreae (incl. Euchresteae and Thermopsideae) needs more careful consideration. The ITS phylogeny suggests that inclusion of *Bolusanthus* and *Dicraeopetalum* into new Sophoreae could make this tribe polyphyletic because those genera reside within a clade only distantly related to other Sophoreae *s*.*s*., and they are grouped with other Afro-Madagascan genera, *Neoharmsia* R. Vig. and *Platycelyphium* [54]. Furthermore, some morphologically disparate characteristics of *Dicraeopetalum*, e.g., radially symmetrical flowers, could make new Sophoreae too complicated to be a natural group. Hence, extensive analyses of morphology and molecular phylogeny based on nuclear as well as plastid data are needed to accomplish formalization of new Sophoreae.

### Legume phylogeny with a “plastome-scale data set”

Recent progress in the sequencing of legume plastomes has provided a great deal of valuable data [30, 32, 45, 55–59] that can be used to make phylogenetic inferences based on a “plastome-scale data set” [29]. Those approaches have become more common since the development of next-generation DNA sequencing [28, 30, 60]. Moreover, the usefulness of plastomes in phylogenetic analyses has proven to show good resolution among evolutionarily puzzling taxa [17]. In our study, we examined 43 legumes belonging to 22 tribes, based on 71 conserved protein-coding genes in their plastomes (Fig 6). Previously, the plastome-scale phylogeny that featured the most comprehensive sampling for legumes had been made by Schwarz et al. [45]. They included 32 plastomes belonging to 16 tribes. When one considers the wide diversity inherent to legumes, our sample size was still small. Most tribes (14 of 22) have not been sufficiently sampled to test monophyly. Nevertheless, our ML analysis produced a phylogenetic tree that is very representative of the current understanding about legume phylogeny among higher taxa [2]. This is certainly true for the monophyly of informal groups and the non-monophyly of some legume tribes. For example, large informal taxonomic groups of Papilionoideae (dalbergioid *s*.*l*., genistoid, indigoferoid/millettioid, robinioid, and IRLC) are resolved as a monophyletic group with 100% bootstrap support. However, the subfamily Caesalpinioideae and six of the 22 legume tribes are not monophyletic (i.e., Caesalpinieae, Mimoseae, Millettieae Phaseoleae, Galegeae, and Trifolieae). In that sense, we believe it is notable that our phylogenetic analysis supports genistoids as sister to the Old World clade and dalbergioid as an earlier diverging lineage. Although the individual groupings of dalbergioid and genistoids are well-established, their phylogenetic positions and interrelationship are still ambiguous. The topology we describe here may have resulted from taxon sampling bias, due to a lack of enough basal papilionoids. However, the high bootstrap values and overall concordance in topology of Old World clades reported previously in other published studies at least suggest that additional plastome sequences for basal papilionoids will be promising data for investigating the higher-level systematics of legumes.

## Conclusion

The first plastome from a member of the tribe Sophoreae *s*.*s*. (*Maackia fauriei*) that we have presented here illustrates the additional, independent loss of *rps16* genes from genistoids. Along with the recent discovery of a 36-kb inversion [32], the novel 24-kb inversion described here is critical evidence of the systematics of genistoids and new Sophoreae sensu Cardoso et al. [4]. Our plastome phylogeny demonstrates its potential usefulness when investigating early-diverging groups of Papilionoideae. Thus, sequence variations and the structural rearrangement of these plastomes will serve as a powerful marker when making formal taxonomic treatments for currently known non-monophyletic tribes of legumes and, most importantly, the new Sophoreae of genistoids.
